# Supplementary material for: Multi-model functionalization of disease-associated PTEN missense mutations identifies multiple molecular mechanisms underlying protein dysfunction
Source: Nat Commun. 2020 Apr 29;11:2073. doi: 10.1038/s41467-020-15943-0 (PMC7190743; doi:10.1038/s41467-020-15943-0)
Supplement: Supplementary file 1 — Supplementary Information [file 41467_2020_15943_MOESM1_ESM.pdf]

SUPPLEMENTARY FIGURES

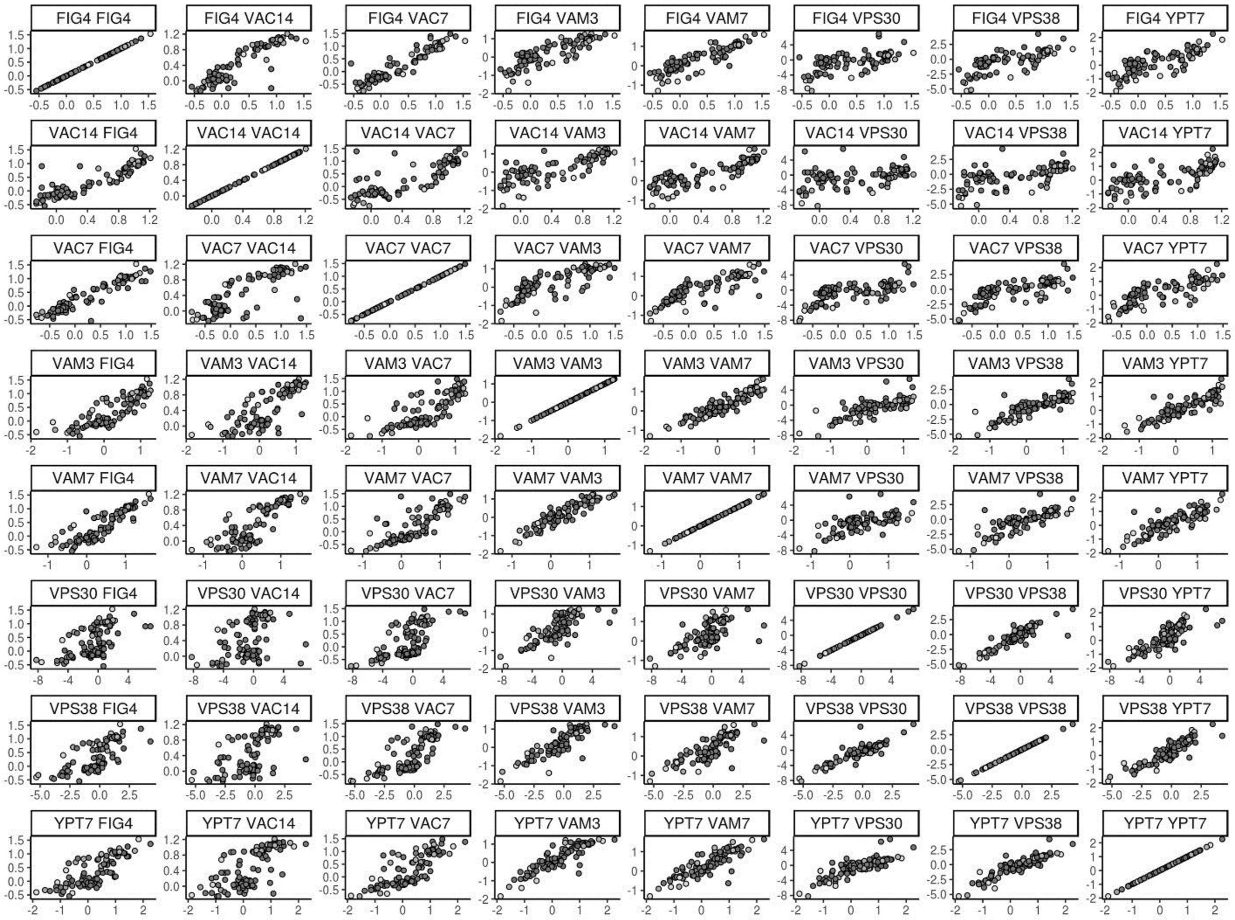

Supplementary Figure 1. **Correlations between 8 sentinel yeast strains.** Correlation plot matrix across all eight yeast sentinels assayed for genetic interaction with PTEN. Pearson coefficients and p values in **Supplementary Table 3**. Source data are provided as a Source Data file.

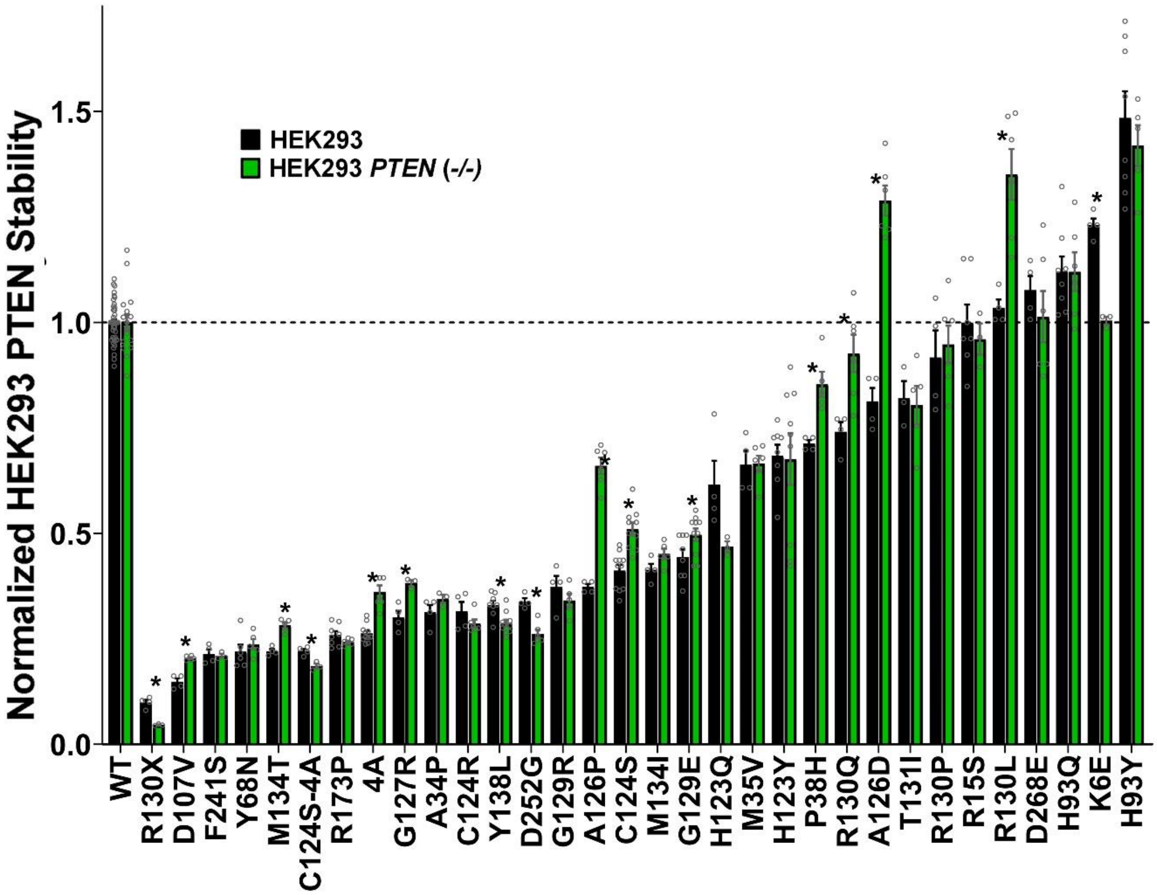

Supplementary Figure 2. Little impact of PTEN KO on exogenous PTEN variant stability. Protein stability of 32 PTEN variants assayed in parental HEK293 cells (black) and HEK293 PTEN KO cells (green) by flow cytometry calculated as median sfGFP/mTagRFPt and expressed as normalized to WT = 1. Data are expressed as mean of well replicates ±SEM. Values are normalized to WT=1 and no difference to untransfected=0. * indicates nominal $p<0.05$ comparing each variant in parental and a PTEN-KO HEK293 cell line by two-tailed Student's t-test. Source data are provided as a Source Data file.

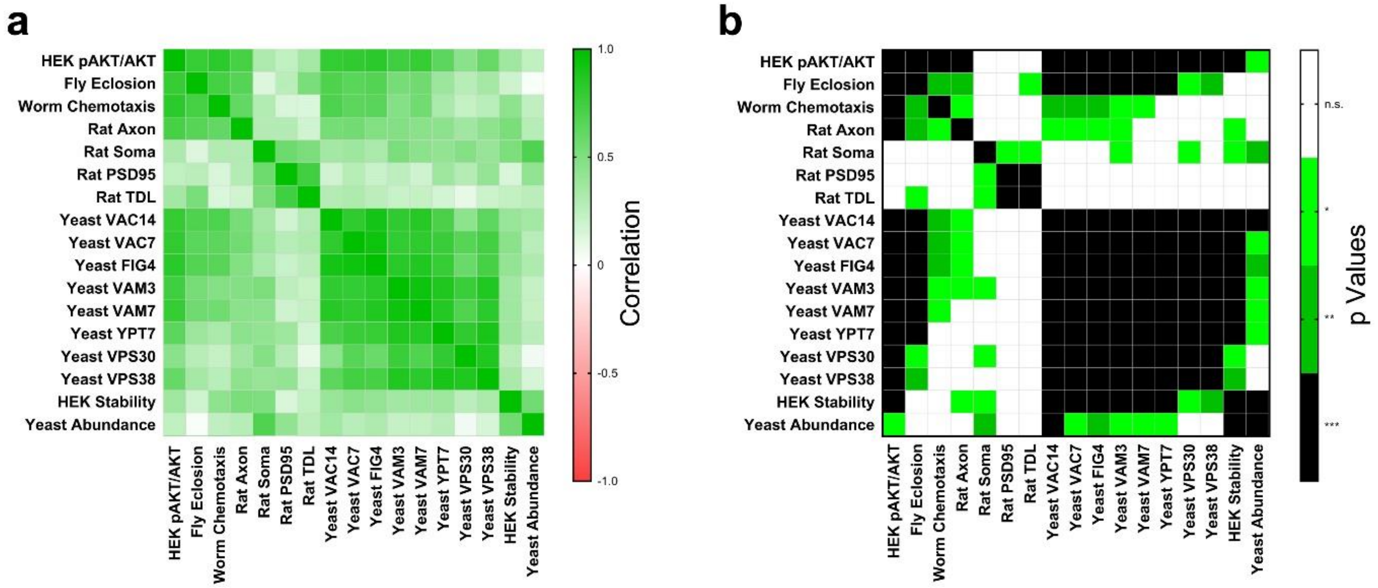

Supplementary Figure 3. Correlations between 17 assays in 5 model systems. a Pearson correlation matrix across model-systems and assays for all variants assayed in each dataset, with color scale at right. Strong correlation of the two stability/abundance datasets in yeast and HEK293 with functional metrics across assays. **b** p-value matrix plot of Pearson correlation matrix indicating different significance thresholds, with color scale at right. Source data are provided as a Source Data file.

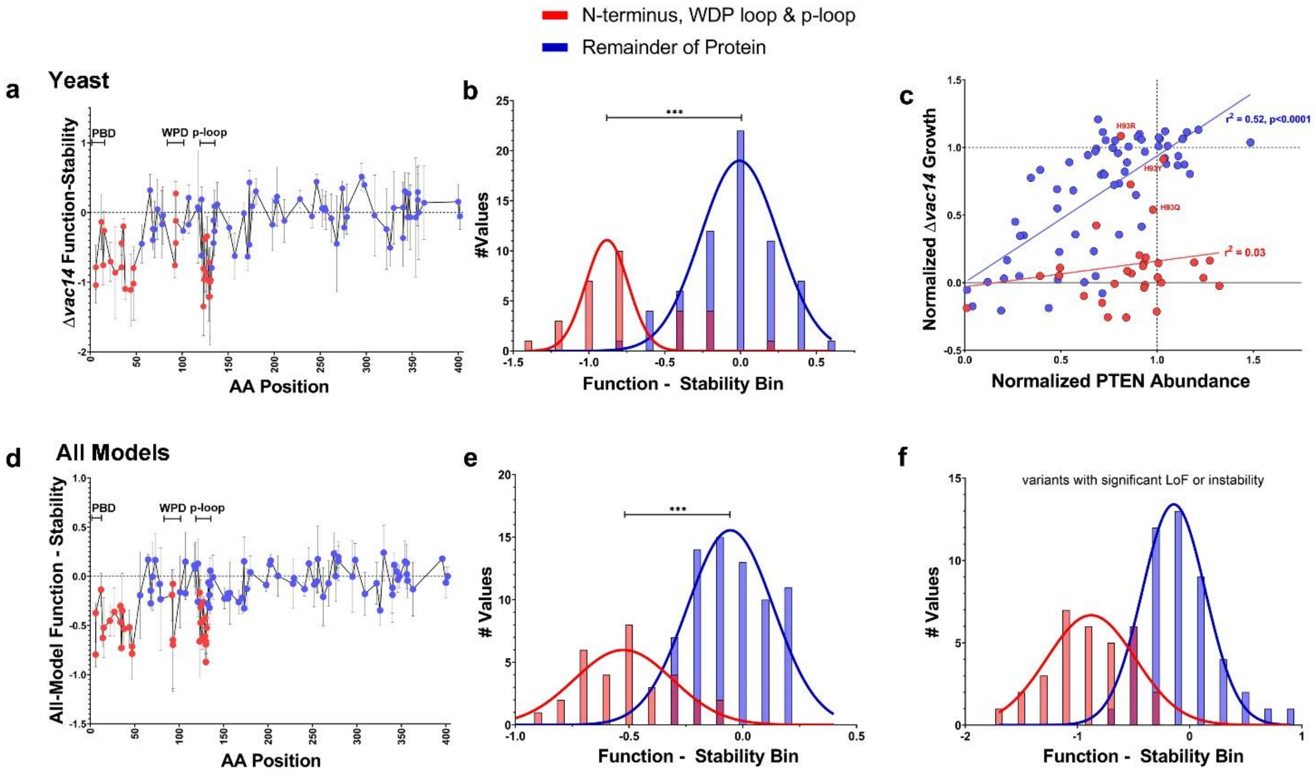

Supplementary Figure 4. Stability-dependent and –independent PTEN domains. a Average variant function-stability (nF – nS) scores for the yeast *Δvac14* assay plotted against amino acid (AA) position, showing clear separation of variants with low scores (stability-independent) predominantly in the well-characterized domains within the N-terminus, WPD-loop and P-loop (red), and variants exhibiting nF – nS ≈ 0 outside these domains (blue). Abundance in yeast used for nS. Data are expressed as means ±*SEM*. **b** Frequency distribution of nF – nS for the yeast *Δvac14* assay showing two distinct distributions for variants in stability-independent (red) and –dependent (blue) domains. *** indicates nominal p<0.0001 by two-tailed Student's t test. **c** Yeast *Δvac14* assay data plotting PTEN variant function vs. abundance with data separated by variants within stability-independent (red) and –dependent (blue). **d** Average variant nF – nS scores (using yeast abundance for nS for yeast, and HEK293 stability for all other models) across all assays plotted against AA position with variants separated into variants in stability-independent (red) and –dependent (blue) domains. **e** Frequency distribution of nF – nS for all assays showing two distinct distributions for variants in stability-independent (red) and –dependent (blue) domains. Data are expressed as means ±SEM. **f** Frequency distribution of nF – nS for all assays as in (**e**), but with removing variants exhibiting WT-like function or stability *** indicates nominal p<0.0001 by two-tailed Student's t test. Individual variant means, error, and n for all plots are provided in the Source Data file.

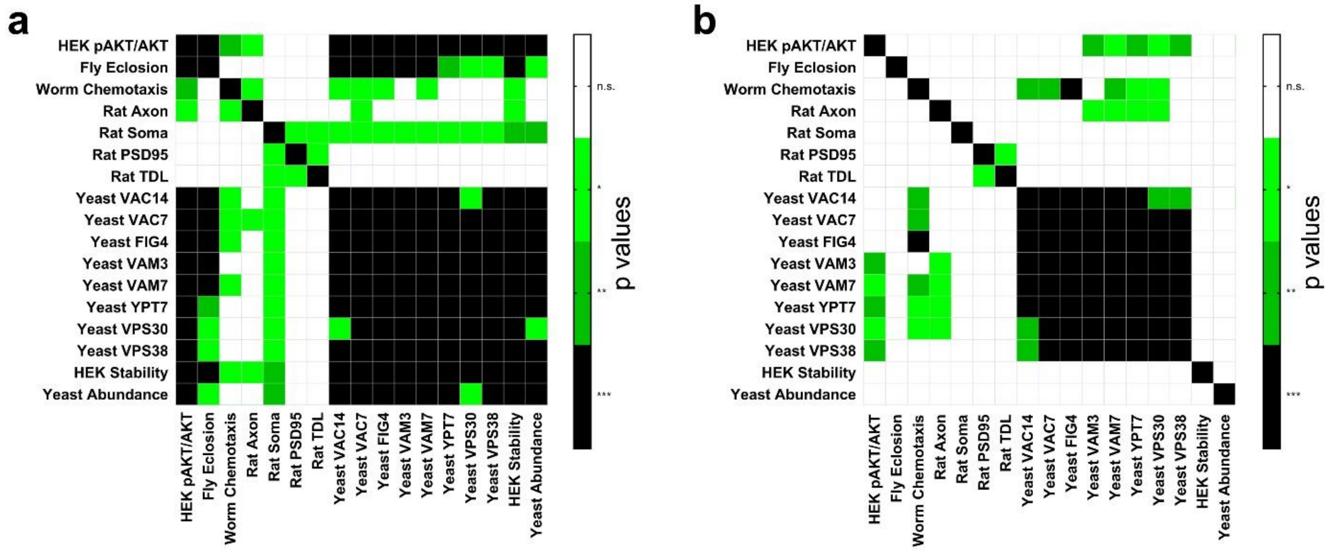

Supplementary Figure 5. P-vaules associated with Figure 6. a,b p-value matrix plots of Pearson correlation matrices shown in **Fig.s 6e,f** indicating stronger significance between assays for variants in stability-dependent (**a**) compared to –independent (**b**) domains. Color scales at right of each figure. Data values are provided a a Source Data file.

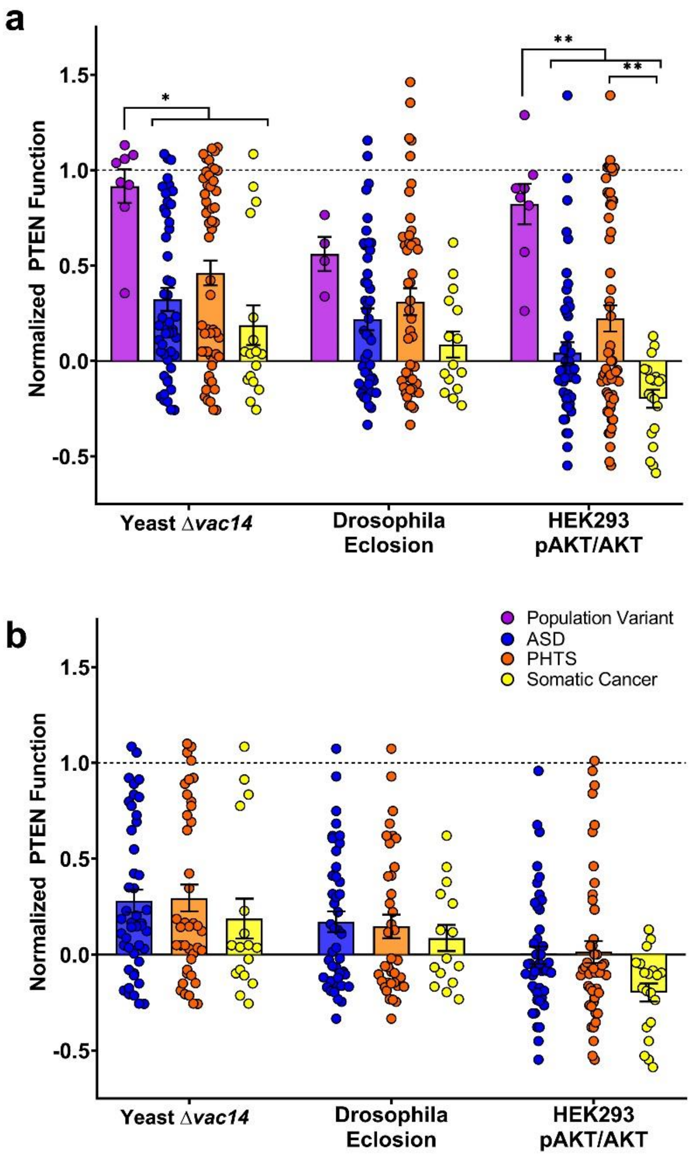

Supplementary Figure 6. PTEN variant dysfunction does not predict disease state. a Distribution of normalized PTEN variant function by associated disease phenotypes in three high-throughput functional assays. Population variants – purple, ASD – blue, PHTS orange, and somatic cancer – yellow. Variants within each phenotype subgroup are not mutually exclusive as several have been found in multiple syndromes/conditions (see **Fig. 6a**). **b** Data as in (**a**), removing variants we predict to be benign (**Fig. 8b**). Mean PTEN function is plotted as bars ± SEM. * indicates $p<0.05$, ** indicates $p<0.005$ by two-way ANOVA. Data values and n's are provided in the Source Data file.

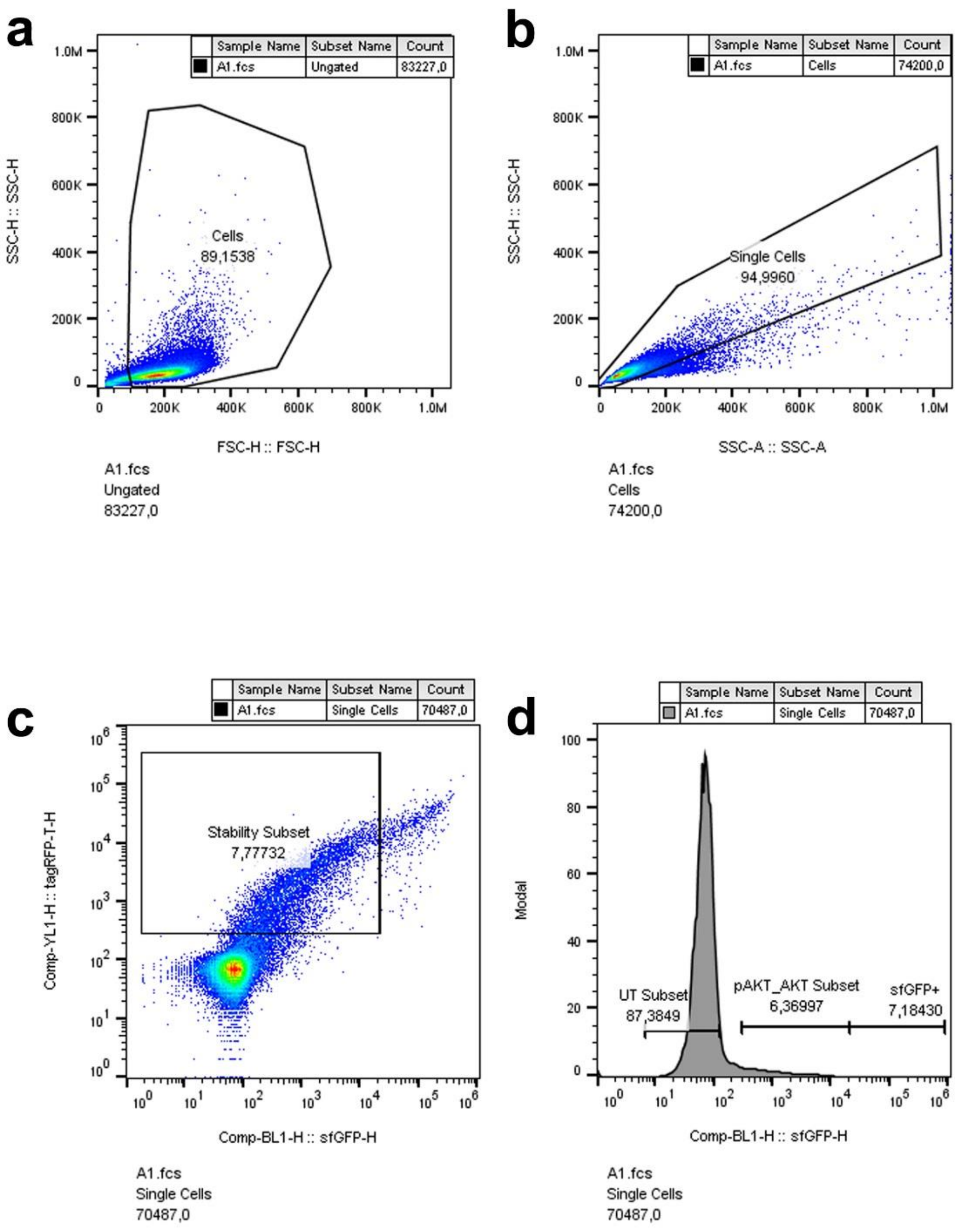

Supplementary Figure 7. Gating Strategy for Flow Cytometry. a Cells were selected from FSC-H vs SSC-H (~90% of events). **b** Singlets were selected from SSC-A vs SSC-H (~90% of cells). **c** Cells for Stability measurement (Stability Subset) were taken from RFP+ cells, excluding ultra-bright (>100-fold over UT) GFP cells (~8% of Singlets). **d** Cells for pAKT/AKT measurement were taken from GFP+ cells, excluding ultra-bright GFP cells (~6% of Singlets).

SUPPLEMENTARY TABLE 1. *C. elegans* strains created for this study.

VG667-668, 672-673 *yvEx667-668, 672-673[paex-3::PTEN::unc-54; pmyo-2::mCherry::unc-54 UTR]*
VG674, 810-816 *daf-18(e1375); yvEx674, 810-816[paex-3::PTEN::unc-54; pmyo-2::mCherry::unc-54 UTR]*
VG723-724 *daf-18(e1375); yvEx723-724[paex-3::PTEN-*C124S*::unc-54; pmyo-2::mCherry::unc-54 UTR]*
VG718-719 *daf-18(e1375); yvEx718-719[paex-3::PTEN-*G132D*::unc-54; pmyo-2::mCherry::unc-54 UTR]*
VG716-717 *daf-18(e1375); yvEx716-717[paex-3::PTEN-*Y176C*::unc-54; pmyo-2::mCherry::unc-54 UTR]*
VG720-721,722-723 *daf-18(e1375); yvEx720-721[paex-3::PTEN-*H123Q*::unc-54; pmyo-2::mCherry::unc-54 UTR]*
VG718-719 *daf-18(e1375); yvEx718-719[paex-3::PTEN-*G132D*::unc-54; pmyo-2::mCherry::unc-54 UTR]*
VG725-726 *daf-18(e1375); yvEx725-726[paex-3::PTEN-I101T::unc-54 UTR; pmyo-2::mCherry::unc-54 UTR]*
VG727, 762-764 *daf-18(e1375); yvEx727, 762-764[paex-3::PTEN-D326N::unc-54 UTR; pmyo-2::mCherry::unc-54 UTR]*
VG729-731 *daf-18(e1375); yvEx729-731[paex-3::PTEN-D92N::unc-54 UTR; pmyo-2::mCherry::unc-54 UTR]*
VG732-733, VG752-753 *daf-18(e1375); yvEx732-733, 752-753[paex-3::PTEN-R130L::unc-54 UTR; pmyo-2::mCherry::unc-54 UTR]*
VG754, 756 *daf-18(e1375); yvEx754-756[paex-3::PTEN-P38H::unc-54 UTR; pmyo-2::mCherry::unc-54 UTR]*
VG758-761 *daf-18(e1375); yvEx758-761[paex-3::PTEN-T167N::unc-54 UTR; pmyo-2::mCherry::unc-54 UTR]*
VG765-768 *daf-18(e1375); yvEx765-768[paex-3::PTEN-T131I::unc-54 UTR; pmyo-2::mCherry::unc-54 UTR]*
VG769-772 *daf-18(e1375); yvEx69-772[paex-3::PTEN-H93R::unc-54 UTR; pmyo-2::mCherry::unc-54 UTR]*
VG773-776 *daf-18(e1375); yvEx773-776[paex-3::PTEN-D268E::unc-54 UTR; pmyo-2::mCherry::unc-54 UTR]*
VG777-779 *daf-18(e1375); yvEx777-779[paex-3::PTEN-G44D::unc-54 UTR; pmyo-2::mCherry::unc-54 UTR]*
VG780-785 *daf-18(e1375); yvEx780-785[paex-3::PTEN-Q171E::unc-54 UTR; pmyo-2::mCherry::unc-54 UTR]*
VG792-795 *daf-18(e1375); yvEx792-795[paex-3::PTEN-K6E::unc-54 UTR; pmyo-2::mCherry::unc-54 UTR]*
VG796-798 *daf-18(e1375); yvEx796-798[paex-3::PTEN-A79T::unc-54 UTR; pmyo-2::mCherry::unc-54 UTR]*
VG805-806 *daf-18(e1375); yvEx805-806[paex-3::PTEN-K6I::unc-54 UTR; pmyo-2::mCherry::unc-54 UTR]*
VG807-809 *daf-18(e1375); yvEx807-809[paex-3::PTEN-P354Q::unc-54 UTR; pmyo-2::mCherry::unc-54 UTR]*

SUPPLEMENTARY DATA 1. Annotation of PTEN variants.

SUPPLEMENTARY DATA 2. Pearson coefficient r and p-values for comparisons between all assays.

SUPPLEMENTARY DATA 3. Primers used to create PTEN variants.
