# Supplementary material for: Multi-model functionalization of disease-associated PTEN missense mutations identifies multiple molecular mechanisms underlying protein dysfunction
Source: Nat Commun. 2020 Apr 29;11:2073. doi: 10.1038/s41467-020-15943-0 (PMC7190743; doi:10.1038/s41467-020-15943-0)
Supplement: Supplementary file 3 — Description of Additional Supplementary Files [file 41467_2020_15943_MOESM3_ESM.pdf]

Title: SUPPLEMENTARY DATA 1. Annotation of PTEN variants.

Description: Each PTEN variant used in this study is listed with its categorization employed here, predicted impact on protein dysfunction from SNAP2 and CADD phred, whether the variant has been identified as *de novo*, found in an individual with ASD, DD, somatic cancer, or PHTS, frequency in the gnomAD and ExAV databases, and clinical assessment.

Title: SUPPLEMENTARY DATA 2. Pearson coefficient r and p-values for comparisons between all assays.

Description: Correlation values are provided for all assays including genetic interactions with 8 yeast sentinel strains. Values are highlighted in green for $p<0.05$.

Title: SUPPLEMENTARY DATA 3. Primers used to create PTEN variants.

Description: For each PTEN variant tested in this study, we provide the primer pairs used in their construction.
