# Supplementary material for: Multi-model functionalization of disease-associated PTEN missense mutations identifies multiple molecular mechanisms underlying protein dysfunction
Source: Nat Commun. 2020 Apr 29;11:2073. doi: 10.1038/s41467-020-15943-0 (PMC7190743; doi:10.1038/s41467-020-15943-0)
Supplement: Supplementary file 7 — Reporting Summary [file 41467_2020_15943_MOESM7_ESM.pdf]

nature research

Corresponding author(s): Kurt Haas, Paul Pavildis

Last updated by author(s): Mar 11, 2020

Reporting Summary

Nature Research wishes to improve the reproducibility of the work that we publish. This form provides structure for consistency and transparency in reporting. For further information on Nature Research policies, see Authors & Referees and the Editorial Policy Checklist .

Statistics

For all statistical analyses, confirm that the following items are present in the figure legend, table legend, main text, or Methods section.

n/a	Confirmed	
☐	☒	The exact sample size (*n*) for each experimental group/condition, given as a discrete number and unit of measurement
☐	☒	A statement on whether measurements were taken from distinct samples or whether the same sample was measured repeatedly
☐	☒	The statistical test(s) used AND whether they are one- or two-sided<br>*Only common tests should be described solely by name; describe more complex techniques in the Methods section.*
☐	☒	A description of all covariates tested
☐	☒	A description of any assumptions or corrections, such as tests of normality and adjustment for multiple comparisons
☐	☒	A full description of the statistical parameters including central tendency (e.g. means) or other basic estimates (e.g. regression coefficient) AND variation (e.g. standard deviation) or associated estimates of uncertainty (e.g. confidence intervals)
☐	☒	For null hypothesis testing, the test statistic (e.g. *F*, *t*, *r*) with confidence intervals, effect sizes, degrees of freedom and *P* value noted<br>*Give P values as exact values whenever suitable.*
☒	☐	For Bayesian analysis, information on the choice of priors and Markov chain Monte Carlo settings
☒	☐	For hierarchical and complex designs, identification of the appropriate level for tests and full reporting of outcomes
☐	☒	Estimates of effect sizes (e.g. Cohen's *d*, Pearson's *r*), indicating how they were calculated

Our web collection on statistics for biologists contains articles on many of the points above.

Software and code

Policy information about availability of computer code

Data collection	Code for data analysis from Pavlidis Lab: https://github.com/PavlidisLab/Post-PTEN<br>Balony Version 1.4.5: https://github.com/barrypyoung/balony/releases<br>TransVar 2.4.0: https://bioinformatics.mdanderson.org/public-software/transvar/
Data analysis	Rat: ImageJsion Version 1.52t: https://imagej.nih.gov/ij/<br>HEK293 Flow cytometry: FlowJo Version 10<br>Data: GraphPad Prism Version 8;<br>Microsoft Excel 2016;<br>R 3.5;<br>RStudio 1.1;<br>github.com (https://github.com/PavlidisLab/Post-PTEN);<br>SNAP2 (predictions computed from the proteome UP000005640 as of October 2015, source: https://rostlab.org/~tmhpred/<br>UP000005640_9606_snap2.tar.bz2);<br>Annovar (version 2016-02-01, build hg19, protocols: refGene,exac03,ljb26_all,cadd13gt10,clinvar_20150629);<br>wAnnovar (http://wannovar.wglab.org/, public interface accessed up to June 2019, builds GRCH37/hg19);<br>TransVar (https://bioinformatics.mdanderson.org/transvar/, public interface accessed up to June 2019, builds GRCH37/hg19)

For manuscripts utilizing custom algorithms or software that are central to the research but not yet described in published literature, software must be made available to editors/reviewers. We strongly encourage code deposition in a community repository (e.g. GitHub). See the Nature Research guidelines for submitting code & software for further information.

Data

Policy information about availability of data

All manuscripts must include a data availability statement. This statement should provide the following information, where applicable:

- Accession codes, unique identifiers, or web links for publicly available datasets
- A list of figures that have associated raw data
- A description of any restrictions on data availability

This work makes use of the following publicly available databases:
VariCarta1 (https://varicarta.msl.ubc.ca);
SFARI Gene (https://gene.sfari.org/);
ClinVar (https://www.ncbi.nlm.nih.gov/clinvar/);
COSMIC (https://cancer.sanger.ac.uk/cosmic);
ExAC (http://exac.broadinstitute.org/);
gnomAD v2.1.1 (https://gnomad.broadinstitute.org/);
CADD phred version 1.0 31 (https://cadd.gs.washington.edu/);
SNAP2 (https://www.rostlab.org/services/snap/);
TransVar (https://bioinformatics.mdanderson.org/transvar/);
RefSeq (https://www.ncbi.nlm.nih.gov/refseq/);
Annovar (https://doc-openbio.readthedocs.io/projects/annovar/en/latest/);
wAnnovar (http://wannovar.wglab.org/).

All study data are available from the authors at https://doi.org/10.5683/SP2/DQOKPB. The source data underlying Fig.s 1-8, and Supplementary Fig.s 1-6 are provided as a Source Data file.

Field-specific reporting

Please select the one below that is the best fit for your research. If you are not sure, read the appropriate sections before making your selection.

☒ Life sciences ☐ Behavioural & social sciences ☐ Ecological, evolutionary & environmental sciences

For a reference copy of the document with all sections, see nature.com/documents/nr-reporting-summary-flat.pdf

Life sciences study design

All studies must disclose on these points even when the disclosure is negative.

Sample size

For Yeast: Sample size for the mini array was selected based on pinning design and plate dimensions. For design, we chose to test 7 variants plus empty vector compared to PTEN-WT on each 1536 plate. Because we had eight sentinels to test, this allowed us to express each variant-WT pair in 12 replicates per plate. The large sample size of the mini arrays is also to account for positional effects and any failure of pinning due to irregularities in plates or pads.

For Drosophila: We only considered vials which had between 10-60 total progeny eclose; all others were excluded from analysis. These numbers were selected to: (i) control for the condition of the vial (<10 flies eclosed would suggest poor food quality or any other vial condition issue), (ii) control for crowding in the vial which may artificially disadvantage PTEN expressing individuals (>60 flies eclosing from a vial would suggest overcrowding of larvae in the vial). Our preliminary testing determined a number of parameters for our eclosion assay protocols: (i) We ascertained the number of male and female adults required and the appropriate egg laying time to reproducibly obtain 10-60 eclosing progeny per vial. (ii) We ascertained how many vials (replicates) to set up in order to efficiently determine variant function. We found that three independent vials with a different set of adults, crossed on different days, was sufficient to assess variant function.

For HEK293 Flow Cytometry: We employed an N>=100 cells per replicate.

For Rat: No statistical analysis was used to predetermine the sample sizes used for our experiments; however the sample sizes are consistent with published literature from our lab and others in the field (eg at least 8-15 cells per culture over at least 3 cultures to give 24-45 cells per condition for analysis; Thomas et al., 2012; Fukata et al., 2013; Brigidi et al., 2014,2015; Shah et al., 2019; Shimell et al., 2019). In the case of the control, human WT , and C124S the numbers are inflated due to transfecting and analyzing for each replicate to ensure biological validity.

For C. elegans: For C. elegans no sample size calculations were performed. Sample sizes were chosen to be larger than those previously used to detect biologically meaningful differences in the literature. Refs: (https://www.ncbi.nlm.nih.gov/pubmed/16950159), (https://dmm.biologists.org/content/11/12/dmm036517.abstract)

Data exclusions

Some variants of PTEN were assayed but not included in final study because clinical annotation was too poor to use or they were removed from clinical databases (e.g. gnomAD/ClinVAR) at a later time. In the model organism assays, a list of excluded variants and label correction from the raw data can be found at https://github.com/PavlidisLab/Post-PTEN/blob/master/RawData/README.md

For the yeast protein abundance assay we did not include data for variant Q396R because we unable to detect this variant on a western using a PTEN antibody, although we detected GFP fluorescence when expressing the GFP-fusion of this variant in our HEK293 flow cytometry stability assay. Since we could detect this fusion with a GFP antibody, but not the PTEN antibody, and the PTEN antibody was made against the C-terminal domain, we conclude that Q396R is located within, and disrupts this antibody's epitope.

Replication

For Yeast: Our synthetic dosage lethality (SDL) screen were performed in biological triplicate. Two independent PTEN-WT SDL screens were performed, therefore there was an n of 6. The attempts of these screens were all successful, however, a few individual strains did not survive to the completion of each experiment. This is not unexpected because some of the deletion strains are slow growing and not easily amenable to the SDL assay regardless of query gene expression. Additionally, variation in pinning pads and plates can influence whether or not a colony is successfully transferred. For the mini array, all PTEN variants are expressed in all sentinels 12 times (biological replicates).

For Drosophila: For the fly assays, all variants were randomly assayed within one of 10 groups of fly crosses. For every one of these groups, we also included (replicated) PTEN-C124S, PTEN WT and attp2 genotypes. In addition, we randomly replicated the assay for 6 variants from different groups. In all cases, we always observed similar data for each variant. Only in one case did we reject an entire group (prior to assaying eclosion time), and repeat the group, because the incubator used (set to 25oC, 75% humidity and a 12hr light/dark cycle) had a humidity fluctuation mid experiment.

For HEK293 Flow Cytometry: data was replicated using different DNA preparations for transfection, transfection performed using different transfection mixes on different days with cell lines at different passage numbers.

For Rat: All experiments for hippocampal neurons were replicated over at least 3 independent cultures. All analyzed replicates were successful, and experiments were only omitted if cell health was deemed subpar (by high numbers of cell death and/or highly variable control cells).

For C. elegans: All Multi-Worm Tracker experiments were replicated at least twice. All replication attempts with successful control groups were successful.

Randomization

For Yeast: Allocation into experimental or control groups was random.

For Drosophila: Allocation was random.

For HEK293 Flow Cytometry: Allocation into experimental or control groups was random.

For Rat: This is not relevant to our study. All samples were prepared from hippocampi dissected from embryonic day 18 (E18) Sprague-Dawley rat pups of either sex and plated at a density of 130 cells/mm2. The protocol used creates a relatively homogenous population of hippocampal pyramidal neurons with minimal glial cell and interneuron contamination, leading to excellent reproducibility and valid for the investigation of cellular mechanisms including (but not limited to) immunohistochemical analysis, morphological characterization, and molecular/biochemical manipulations.

For C. elegans: C. elegans were partitioned into group based on their genotype. All other covariates (e.g. age, growth conditions, etc.) were held equal.

Blinding

For Yeast: All grow assays were performed with experimenter blinded to variant.

For Drosophila: For every variant we assayed, a number code (unrelated to actual variant identity) was assigned by the transgenic company. The experimenter was not provided variant identity (just that number code) until after data was analyzed. The PTEN C124S, PTEN WT and attP2, were repeated in every group; the experimenter was further blinded to these during data collection. All raw data for statistical analysis was conducted by a different investigator.

For HEK293 Flow Cytometry: Blinding was employed by using automated data collection and processing and standardized for all samples and replicates, with annotation/identification of the variant only conducted for statistical analysis in the last step.

For Rat: For hippocampal culture experiments, investigators/experimenters were not blinded to conditions. However, as is common practice in our lab, we had regular validation checks whereby we had other lab members blinded to conditions check a subset of cells to ensure that they were getting similar results.

For C. elegans: No blinding was necessary for C. elegans as the Multi-Worm Tracker scores behavior objectively.

Reporting for specific materials, systems and methods

We require information from authors about some types of materials, experimental systems and methods used in many studies. Here, indicate whether each material, system or method listed is relevant to your study. If you are not sure if a list item applies to your research, read the appropriate section before selecting a response.

Materials & experimental systems

n/a	Involved in the study
[ ]	[x] Antibodies
[ ]	[x] Eukaryotic cell lines
[x]	[ ] Palaeontology
[ ]	[x] Animals and other organisms
[x]	[ ] Human research participants
[x]	[ ] Clinical data

Methods

n/a	Involved in the study
[x]	[ ] ChIP-seq
[ ]	[x] Flow cytometry
[x]	[ ] MRI-based neuroimaging

Antibodies

Antibodies used

For Yeast:
PTEN antibody (1:1000; R&D Systems, cat.#MAB847)
Goat-anti-Mouse HRP (1:5000; Thermo Fisher Scientific, cat.#62-6520)

For HEK293 Flow Cytometry:
Rabbit anti-pAKT (1:100; Cell Signaling, Ser473, cat.#9271)
Mouse anti-pan-AKT (1:100; Cell Signaling, cat.#2920)
Goat anti-Rabbit IgG-Alexa Fluor 647 (1:100; Thermo Fisher, cat.#A21244)
Goat anti-Mouse IgG-Alexa Fluor 405 (1:100; Thermo Fisher, cat#.A31553)

For Rat:
Primary antibodies:
Mouse monoclonal (IgG2a) anti-PSD95 (1:500; Abcam, cat.#ab2723, clone: 6G6-1C9, LOT#GR299294-3, RRID: AB_303248)
Mouse monoclonal (IgG1) anti-Gephyrin (1:300; Synaptic Systems, cat.#147 011, clone: mAb7a, RRID: AB_887717)
Rabbit monoclonal (IgG) anti-HA (Cell Signalling Technology, cat.#3724S, clone C29F4, RRID: AB_1549585)
Mouse monoclonal (IgG2a) anti-Beta-Tubulin III (1:500; STEMCELL Technologies, cat.#60052, clone TUJ1)
Secondary antibodies:
Goat anti-mouse Alexa Fluor 488, (1:1000; ThermoFisher, Molecular Probes, cat.# A-11001; RRID: AB_2534069)
Goat anti-mouse Alexa Fluor 568, (1:1000; ThermoFisher, Molecular Probes, cat.#A-11019; RRID: AB_143162).
Goat Anti-Mouse IgG Texas Red dye-conjugated AffiniPure (1:250; Jackson ImmunoResearch Laboratories Inc., cat.#111-075-144)

Validation

For Yeast:
PTEN antibody (1:1000; R&D Systems, cat.#MAB847) validated for WB, IHC & Flow.
HRP validated for WB, ELISA, ICC & IHC.

For HEK293 Flow Cytometry:
Rabbit anti-pAKT (1:100; Cell Signaling, Ser473, cat.#9271) and Mouse anti-pan-AKT (1:100; Cell Signaling, cat.#2920) primary antibodies were validated by the manufacturer for Flow Cytometry, IF, IP and WB. Both secondary antibodies were validated by the manufacturer for Flow Cytometry, IF and ICC.

For Rat:
Mouse monoclonal (IgG2a) anti-PSD95 (Abcam, cat.#ab2723, clone: 6G6-1C9, LOT#GR299294-3, RRID: AB_303248)
From Abcam: "Ab promise guarantee coves the use of ab2723 in IHC, WB, IP. Host species Mouse. Specificity We do not guarantee IHC-P for mouse. Tested applications Suitable for: IHC-P, WB, IHC-Fr, IP, IHC-FoFr. Species reactivity Reacts with: Mouse, Rat, Zebrafish."99 citations on Abcam website.
The Bamji lab validated this antibody, reported in Brigidi et al., 2015; Shah et al., 2019; Shimell et al., 2019.

Mouse monoclonal (IgG1) anti-Gephyrin (Synaptic Systems, 147 011, clone: mAb7a, RRID: AB_887717)
From Synaptic Systems: "Applications: WB: not recommended; IP: not recommended; ICC: 1 : 250 up to 1 : 1000; IHC: 1 : 500 Reactivity Reacts with: human (Q9NQX3), rat (Q03555), mouse (Q8BUV3), pig, goldfish, zebrafish, chicken. Other species not tested yet. Specific for the brain-specific 93 kDa splice variant. K.O. verified." 46 references for ICC on Synaptic Systems website).
The Bamji lab validated this antibody, reported in Brigidi et al., 2015; Shah et al., 2019; Shimell et al., 2019;

Mouse monoclonal (IgG2a) anti-Beta-Tubulin III (1:500; STEMCELL Technologies, cat.#60052, clone TUJ1): Company Statement: "The TUJ1 antibody reacts with beta-tubulin III, an ~50 - 55 kDa structural protein that is a component of tubulin. Tubulin is the major component of microtubules within the cytoskeleton and is assembled from heterodimers of alpha and beta tubulin subunits. The beta III isoform of tubulin, also known as neuron-specific class III beta-tubulin, is expressed primarily in neurons and is widely used as a marker to distinguish neurons from other cell types."

Goat anti-mouse Alexa Fluor 488, (1:1000; ThermoFisher, Molecular Probes, cat.# A-11001; RRID: AB_2534069) Validated by vendor with verified applications for IHC.

Goat anti-mouse Alexa Fluor 568, (1:1000; ThermoFisher, Molecular Probes, cat.#A-11019; RRID: AB_143162) Validated by vendor with verified applications for IHC.

Goat Anti-Mouse IgG Texas Red dye-conjugated AffiniPure (1:250; Jackson ImmunoResearch Laboratories Inc., cat.#111-075-144): Whole IgG goat affinity-purified antibodies. Company Statement: "Based on immunoelectrophoresis and/or ELISA, the antibody reacts with whole molecule mouse IgG. It also reacts with the light chains of other mouse immunoglobulins. No antibody was detected against non-immunoglobulin serum proteins."

Eukaryotic cell lines

Policy information about cell lines

Cell line source(s)	HEK293: CRL-1573 from ATCC
Authentication	Not authenticated
Mycoplasma contamination	Not tested
Commonly misidentified lines (See ICLAC register)	No commonly misidentified cell lines were used in the study

Animals and other organisms

Policy information about studies involving animals; ARRIVE guidelines recommended for reporting animal research

Laboratory animals

C. elegans, Drosophila melanogaster, and rat neuronal cultures were used in this study.

For Rats: hippocampal cultures from embryonic day 18 (E18) rat pups of either sex were prepared from timed-pregnant Sprague-Dawley rats (Charles River, Sherbrooke, Canada). Cells were plated on 18 mm coverslips (Marienfeld, Lauda-Konigshofen, Germany) placed in 12 well dishes. The timed-pregnant dams were delivered to the facility and immediately harvested. All experimental procedures and housing conditions were approved by the UBC Animal Care Committee and were in accordance with the Canadian Council on Animal Care (CCAC) guidelines – briefly, the rodent facility where we access the rats is maintained at 20-24°C, 50%±10% relative humidity, and a 12/12 light dark cycle (reversed, ie lights off at 8:00 AM). Upon arrival rodents have ad libitum access to water and food, and timed-pregnant dams are usually sacrificed within an hour of arrival.

For Drosophila: Drosophila melanogaster
daughterless-Gal4, obtained from Auld Lab (UBC).
w1118;; P{w[+mC]=UAS-PTENvar}attP2. Allan lab (plasmid injections by Rainbow Transgenics).
Sex- male and female adult and progeny
Age - all adults crossed at 3-4 days old. Age to eclosion was tracked in progeny by the assay.

For C. elegans: 72-96h old adult hermaphroditic Bristol N2 animals were used as wild-type controls for all experiments. PTEN●daf-18(e1375) mutant worms were used for all transgenesis-based rescue experiments.

Wild animals	No wild animals were used in this study.
Field-collected samples	No field collected samples were used in the study.
Ethics oversight	All experimental procedures and housing conditions were approved by the UBC Animal Care Committee and were in accordance with the Canadian Council on Animal Care (CCAC) guidelines.

Note that full information on the approval of the study protocol must also be provided in the manuscript.

Flow Cytometry

Plots

Confirm that:

- [x] The axis labels state the marker and fluorochrome used (e.g. CD4-FITC).
- [x] The axis scales are clearly visible. Include numbers along axes only for bottom left plot of group (a 'group' is an analysis of identical markers).
- [x] All plots are contour plots with outliers or pseudocolor plots.
- [x] A numerical value for number of cells or percentage (with statistics) is provided.

Methodology

Sample preparation	HEK293 Cells that were trypsinized and fixed in single cell suspension
Instrument	Attune NxT (Invitrogen)

Software	FlowJo Version 10
Cell population abundance	Gating for Cells: ~90%, Gating for Singlets: ~80%, Gating for transfected cells: ~10%
Gating strategy	Cells were selected for Singlets by FSC-H/SSC-H gating followed by SSC-A/SSC-H gating. Untransfected Stained cells were used to determine the cutoff of sfGFP/RFP fluorescence for lower and upper limit of transfected cells. No sorting.

☒ Tick this box to confirm that a figure exemplifying the gating strategy is provided in the Supplementary Information.
